# RUN FOR ANTI-AGING: EXERCISE TIMING SPECIFIES METABOLIC RESPONSES

**DOI:** 10.1093/geroni/igad104.1681

**Published:** 2023-12-21

**Authors:** Shogo Sato

**Affiliations:** Texas A&M University, College Station, Texas, United States

## Abstract

Exercise has been widely recognized as an effective non-pharmacological intervention to delay aging and reduce the onset of chronic metabolic diseases such as type 2 diabetes and cardiovascular diseases. The metabolic response to exercise is influenced by factors such as frequency, intensity, duration, and modality. However, the role of the timing of exercise in activating metabolic pathways has remained an outstanding question. The circadian clock allows the organism to adapt to the cyclic environmental changes imposed by the earth’s rotation, thereby placing temporal boundaries on physiological functions, including metabolic responses to exercise. Our recent studies performed unbiased high-throughput metabolomics of 8 different metabolic tissues in response to a single-bout acute treadmill exercise at different times of the day in mice, revealing that daily timing of exercise is a critical variable for the metabolic impact on different metabolic tissues. This indicates that timing is key to unlocking the benefits of exercise on metabolic health and anti-aging. Therefore, optimizing exercise outcomes following when to exercise is essential to establishing personalized exercise interventions aiming at health improvement and reducing the risk of chronic diseases associated with aging. Our ongoing research is studying the time-of-day-dependent metabolic responses to exercise in aged mice compared to young mice. The ultimate goal is to identify the best time for exercise to promote longevity and healthy aging.

